# Effects of Extrinsic Magnetized GaAs in One-Dimensional Ternary Photonic Crystals

**DOI:** 10.3390/ma18235293

**Published:** 2025-11-24

**Authors:** Amita Biswal, Harekrushna Behera, Tai-Wen Hsu

**Affiliations:** Center of Excellence for Ocean Engineering, National Taiwan Ocean University, Keelung 202301, Taiwan; amitabiswal@mail.ntou.edu.tw

**Keywords:** external magnetic field, ternary photonic crystals, transfer matrix method, lightwave propagation, transmittance spectra

## Abstract

This work focuses on the magneto-optical behavior of one-dimensional ternary photonic crystals that incorporate extrinsically magnetized *GaAs* as a functional layer. In this context, we investigate the effect of an applied transverse magnetic field on the optical response and photonic band gap characteristics of the proposed structure. The transfer matrix method is utilized to analyze the optical response of the ternary structure. The ternary photonic crystal with extrinsically magnetized *GaAs* exhibits strong magnetic tunability. The photonic band gap shifts from 0.32 THz to 0.38 THz under an applied external magnetic field up to 0.75 T with 100% band gap modulation. The polarization mode also shifts within the range of 0.32–0.36/0.38 THz due to the anisotropic response of the magnetized *GaAs*. These results confirm the effectiveness of extrinsic magnetization for compact, dynamically tunable photonic devices. The proposed configuration thus provides an effective framework for developing multichannel and broadband transmission filters that can be adjusted in the terahertz domain.

## 1. Introduction

The idea of photonic crystals (PCs), which control the travel of electromagnetic and optical waves, was initially proposed by Yablonovitch [1]. A defining property of PCs is the formation of photonic band gaps, frequencies in which light cannot propagate due to the periodic arrangement of dielectric materials [2,3,4,5]. Among them, PCs with one dimension (1D) serve as the simplest configuration and are widely regarded as fundamental structures for numerous applications, including light harvesting in solar cells [6], photovoltaic devices [7], high-reflectivity mirrors [8], Fabry–Perot resonators, quantum dot nanowires, and a variety of sensing platforms [9,10,11,12,13,14]. They are also used in hybrid multifunctional devices [15] and optical filters [16]. To tailor and expand the photonic band gap (PBG) over desired spectral ranges, researchers have explored various structural configurations of PCs, including periodic lattices [5], quasi-periodic designs [17,18], random distributions [19,20], and nonlinear nanostructured systems [21].

Over the past few decades, numerous photonic crystal structures have been proposed for diverse applications [22,23,24,25]. Among them, *GaAs/AlAs*-based PCs have gained particular attention because of their distinctive qualities, like their relocation of the infrared spectrum of frequencies (longitudinal optical (LO) and transverse optical (TO)) in the form of a relatively wide band gap [26,27]. The optical behavior of *GaAs/AlAs* compound superlattices with randomly distributed layer thicknesses was analyzed by Chen and Xiong [28] using the Wannier–Bloch mixing technique. Due to the small lattice mismatch of *GaAs* and $Al_{x}Ga_{1-x}As$, this photonic structure is considered the basic concept for the development of advanced high-speed optoelectronic and electronic devices [29].

To achieve advanced multifunctional capabilities of PCs, it is essential to incorporate a controllable material into a perfectly periodic structure [30,31]. This is commonly realized by inserting a single layer into a 1D PC, leading to the emergence of wide-band transmission resonances around the PBG. Extensive research has been devoted to magnetized and disordered 1D PCs; as such, modifications significantly influence transmission characteristics [32]. However, from both fundamental and application-oriented perspectives, challenges remain in precisely engineering extrinsic magnetized PCs beyond the band gap. In particular, the design of externally magnetized states in the visible wavelength regime, where periodicity becomes comparable to optical wavelengths, remains critical for exploring phenomena such as transmission, reflection, and absorption [33,34].

In recent years, all-dielectric photonic crystals engineered with extrinsic materials have attracted significant attention due to their robustness and versatility in practical applications [35,36,37]. Ternary 1D structures are generally realized by continuing the periodic order of a conventional multilayered PC for various medical applications [38,39,40,41], most effectively inserting an extrinsic magnetized layer into each bundle of the periodic lattice [42,43,44]. Incorporating non-linear electro-optical dielectric materials further introduces tunability into such systems for the detection of cancer cells [45]. A notable example is the theoretical study by Taya [46], which demonstrated that adjusting the permittivity of dielectric materials through electro-optical effects can significantly influence the properties of left-handed materials, particularly by enhancing them for refractometric applications.

Most previous studies have focused primarily on analyzing the influence of a single/double layer on the transmission and absorption characteristics of 1D PCs. The proposed configuration exploits the cyclotron resonance–induced permittivity variation in *GaAs* under external transverse magnetic fields, providing a strong and controllable mechanism for PBG tuning without altering the device geometry, and the use of a ternary arrangement further enhances dispersion contrast, enabling pronounced magneto-optical effects even at moderate field strengths. The novelty of our contribution is in the extension and modification of such formulations for a 1D ternary PC containing an extrinsically magnetized *GaAs* layer. The transfer matrix method [47,48] is adapted to handle the anisotropic magneto-optical behavior of *GaAs* in order to compute the field propagation accurately in the presence of an applied transverse magnetic field. We propose a novel ternary structural configuration that is specifically designed to enhance the magneto-optical modulation, distinct from the binary and non-magnetic structures studied previously. We incorporate the field-dependent permittivity model into the optical dispersion relations, which allows us to analyze band gap shifts, as well as transmission modulation for the extrinsic magnetic field, electron density, and incident angle, with different mode splittings (TE and TM).

The structure of the manuscript is as follows: it includes a clear literature study in Section 1, whereas mathematical formulations, the ternary structure, and the transfer matrix method are described in Section 2. Section 3 discusses the numerical results in detail, while the conclusions of the present investigation are summarized in Section 4.

## 2. Methodology and Formulation

**Figure 1:**
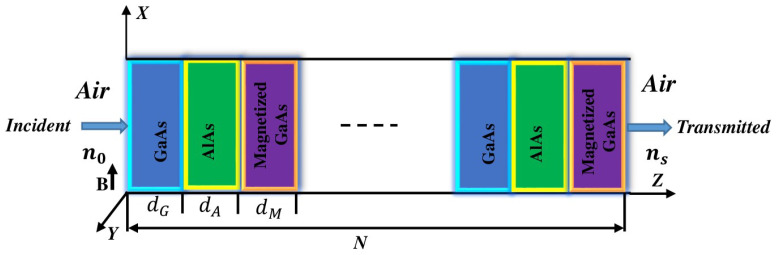
Schematic representation of ternary 1D *GaAs/AlAs*-based photonic crystal containing magnetized *GaAs* as third layers for periodicity (*N*) with external magnetic field (**B**), electron density ($n_{e}$), *GaAs* layer thickness ($d_{G}$), AlAs layer thickness ($d_{A}$), magnetized *GaAs* layer thickness ($d_{M}$), vacuum refractive index ($n_{0}$), and substrate refractive index ($n_{s}$); the entire structure is suspended in air.

The schematic of the proposed one-dimensional ternary photonic crystal, composed of alternating *GaAs/AlAs* layers, incorporating the external magnetic field with *GaAs* as the third dielectric, is illustrated in Figure 1. Further, the incident light enters the structure at an angle $\theta_{0}$ with respect to the *z*-axis and is assumed to propagate normally in both the transverse electric (TE) and transverse magnetic (TM) modes. In the TE mode, the magnetic field is aligned along the propagation direction, whereas in the TM mode, only the electric field exists in that direction [49]. Consequently, the TE and TM modes fully capture the behavior of any arbitrarily polarized wave, which may include circular polarization. Thus, circularly polarized waves do not bring in any extra physics beyond the TE/TM analysis for this 1D structure. The direction of the magnetic field is applied in xz-plane (Figure 1), which results only in diagonal elements of the dielectric tensor [43]. The applied magnetic field is spatially periodic, and therefore maximizes the tunability of the photonic band gap irrespective of the mode. The ternary photonic structure consists of *GaAs*, *AlAs*, and magnetized *GaAs*, whose respective thicknesses are $d_{G}$, $d_{A}$, and $d_{M}$, and whose corresponding permittivities are $\epsilon_{G}$, $\epsilon_{A}$, and $\epsilon_{M}$, respectively.

The governing Maxwell equations and the basic transfer matrix framework are standard and widely used in PC analysis. However, the novelty of our contribution lies in the use of these formulations to extend and modify for the case of a 1D ternary PC containing an extrinsically magnetized *GaAs* layer. In the long-wavelength limit, the complex dielectric permittivity is formulated in Equation (1), as described by Adachi [29].

(1) $$\begin{array}{cc} \; & \epsilon \left(\omega \right)=\epsilon_{\infty }\left\{1+\frac{\omega_{l}^{2}-\omega_{t}^{2}}{\omega_{t}^{2}-\omega^{2}-i\omega \gamma }\right\}, \end{array}$$

where the damping frequency (γ), static dielectric constant ($\epsilon_{\infty }$), and longitudinal and transverse optical frequencies, ($\omega_{l}$ and $\omega_{t}$), respectively, are known as the Reststrahlen parameters. The values of the Reststrahlen parameters for the *GaAs* semiconductor superlattice are $\omega_{l}=291.5$ cm^−1^, $\omega_{t}=268.2$ cm^−1^, γ=2.3 cm^−1^, and $\epsilon_{\infty }=11.1$, whereas $\omega_{l}=361.8$ cm^−1^, $\omega_{t}=1.9$ cm^−1^, γ=8.0 cm^−1^, and $\epsilon_{\infty }=8.2$ are the Reststrahlen parameter values for *AlAs* (see in Adachi [29]).

In the present study, the transmittance characteristics of TE- and TM-polarized waves propagating through the ternary multilayered photonic structure are evaluated using the transfer matrix method. This method provides a systematic approach to relate the electromagnetic field amplitudes at the input and output interfaces of the multilayer system. For a symmetric 1D photonic structure, Equation (2) is the overall transfer matrix, and can be expressed as [47,48]

(2) $$\begin{array}{c} W=\left(\begin{array}{cc} a_{11} & a_{12}\\ a_{21} & a_{22} \end{array}\right)={\left(W_{G}W_{A}W_{M}\right)}^{N} \end{array}$$

Here, the transfer matrices corresponding to different layers are $W_{G}$ for *GaAs*, $W_{A}$ for *AlAs*, and $W_{M}$ for magnetized *GaAs*, respectively. In general, the transfer matrix for the *j*th layer in terms of TE polarization in Equation (3) is $W_{j}$, written as [43]

(3) $$\begin{array}{cc} \; & W_{j}=\left(\begin{array}{cc} \cos(\left(\omega /c\right)n_{j}d_{j}\cos\theta_{j}) & \left(-i/p_{j}\right)\sin\left(\left(\omega /c\right)n_{j}d_{j}\cos\theta_{j}\right)\\ -ip_{j}\sin\left(\left(\omega /c\right)n_{j}d_{j}\cos\theta_{j}\right) & \cos(\left(\omega /c\right)n_{j}d_{j}\cos\theta_{j}) \end{array}\right), \end{array}$$

where *c* denotes the speed of light in vacuum, $p_{j}=\sqrt{\epsilon_{j}/\mu_{j}}\cos\theta_{j}$, $\cos\theta_{j}=\sqrt{1-{\left(n_{0}/n_{j}\right)}^{2}\sin^{2}\theta_{j}}$, $n_{0}$ is the refractive index of the vacuum, and $\theta_{j}$ is the ray angle inside the *i*th layer. Moreover, the refractive index, permeability, permittivity, and thickness of the *j*th layer are denoted by $n_{j}$, $\mu_{j}$, $\epsilon_{j}$, and $d_{j}$, respectively. The transmission coefficient in Equation (4) for the TE-polarized wave is derived by applying the outgoing radiation boundary condition, which can be expressed as

(4) $$\begin{array}{c} t=\frac{2p_{0}}{\left(a_{11}+a_{12}p_{j}\right)p_{0}+\left(a_{21}+a_{22}p_{j}\right)}, \end{array}$$

where $p_{j}=n_{j}\cos\theta_{j}$ with $p_{0}=n_{0}\cos\theta_{0}$. Finally, the transmittance and reflectance corresponding to the multilayered photonic crystal are expressed as $T={\left|t\right|}^{2}$ and $R={\left|r\right|}^{2}=1-T$, respectively, where vacuum is considered at both ends of the PC organization.

Conversely, the transmittance coefficient for the TM-polarized wave is acquired analogously, given by $p_{j}=\cos\theta_{j}/n_{j}$ and $p_{0}=\cos\theta_{0}/n_{0}$ [50]. The absorbance plays a secondary role in our simulations, as extrinsically magnetized *GaAs* exhibits field-dependent complex permittivity. The imaginary part of the permittivity accounts for material absorption, which influences the attenuation of electromagnetic waves and has negligible effects on the transmission and reflection spectra (as in [29]).

## 3. Numerical Results and Discussion

This section presents the numerical results illustrating the influence of various physical parameters on the propagation of electromagnetic waves through a one-dimensional ternary multilayer photonic structure. The parameter values adopted from Arregui et al. [20] and Adachi [29] remain constant throughout the computations unless otherwise specified. Table 1 represents the parametric values used for numerical simulation. In the present model, a total of N=30 layers, including the externally magnetized *GaAs*, are chosen, as this allows for sufficient periodicity to obtain well-defined photonic band gaps and stable polarized modes without introducing unnecessary structural complexity, which keeps the computational cost efficient.

The analysis focuses primarily on the transmission coefficient, T, as a function of frequency, ω, for a 1D ternary photonic structure. The numerical results reveal a noticeable shift in the transverse optical ($\omega_{t}$) and longitudinal optical ($\omega_{l}$) mode frequencies induced by the presence of an external magnetic field. This study considers a periodic structure that incorporates layers of magnetized *GaAs*, which exhibit more band gaps in the near-infrared area.

The results are presented below, demonstrating that extrinsically magnetized GaAs allows for strong magnetic field-dependent tuning in the proposed ternary PC. The anisotropic permittivity of magnetized GaAs introduces band gap shifts, transmission modulation, and band gap mode splitting, which enhance refractive index contrast under an applied field. Compared with the earlier binary or non-magnetic structures, substantially improved tunability is obtained for the present design without any geometrical change. These results confirm the potential of magnetized GaAs for compact magnetically controlled photonic devices.

### 3.1. Transmission Spectra with and Without Ternary Layer

**Figure 2:**
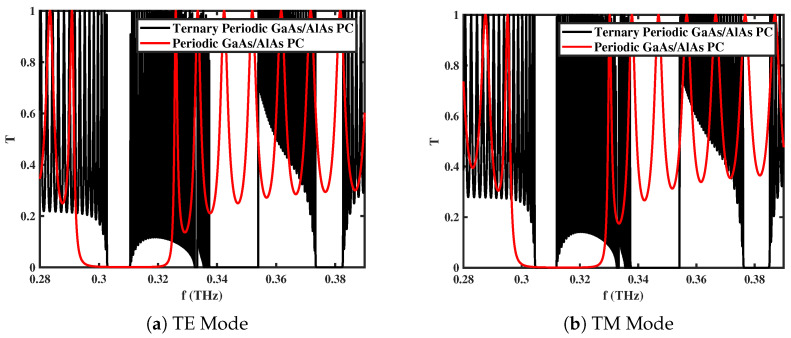
Transmittance spectra of *GaAs/AlAs*-based 1D ternary multilayered PC for different modes of propagation for N=30 with **B** = 0.75 T, $n_{e}=1.5\times 10^{22}$ cm^−3^, and $\theta_{0}=0^{\circ }$.

Figure 2a,b illustrate the transmittance spectra of the TE and TM modes for the 1D *GaAs/AlAs*-based ternary PC with and without magnetized *GaAs* layers. The analysis is performed for N=30 layers under an externally applied magnetic field of **B** = 0.75 T, electron density of $n_{e}=1.5\times 10^{22}$ cm^−3^, and normal incident angle. In the absence of magnetized layers, a single PBG is observed throughout the range of frequency from 0.3 to 0.32 THz (red curve). When the magnetized *GaAs* layers are introduced, three distinct band gaps (black curves) emerge for both polarization modes. These three gaps exhibit complete transmission due to resonance-induced impedance matching [20,43], which occurs when the multilayer structure is assumed to be surrounded by air, resulting in total zero reflection. Hence, the presence of magnetized layers not only broadens the photonic band gaps but also introduces a frequency-dependent complete transmittance, making the structure highly suitable for designing tunable wide-band transmission filters. Such filters can be readily engineered for operation across various frequency ranges according to specific design requirements.

### 3.2. Effect of External Magnetic Field (**B**)

The permittivity of the material can be calculated by applying an external magnetic field (as in [43]).

(5) $$\begin{array}{cc} \; & \epsilon_{A}\left(\omega ,B\right)=\epsilon_{s}\left\{1-\frac{\omega_{p}^{2}\left(\omega^{2}-\omega_{p}^{2}\right)}{\omega^{2}\left(\omega^{2}-\omega_{c}^{2}-\omega_{p}^{2}\right)}\right\}, \end{array}$$

where plasma frequency $\omega_{p}={\left(\frac{n_{e}e^{2}}{m\epsilon_{0}}\right)}^{1/2}$ and the cyclotron frequency $\omega_{c}=\frac{eB}{m^{*}}$, considering the static dielectric constant ($\epsilon_{s}$), electron density ($n_{e}$), electronic charge (*e*), free electron mass (*m*), effective mass of an electron ($m^{*}$), and vacuum permittivity ($\epsilon_{0}$) [19].

**Figure 3:**
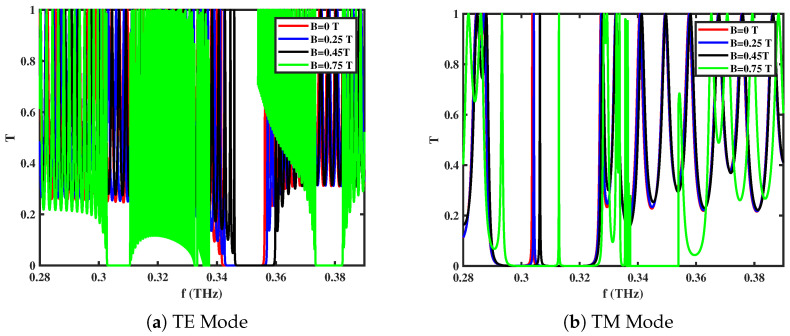
Transmittance spectra of *GaAs/AlAs*-based 1D ternary multilayered PC for external magnetic field (**B**) with $n_{e}=1.5\times 10^{22}$ cm^−3^ and $\theta_{0}=0^{\circ }$ in different modes of propagation.

Figure 3a,b illustrate the influence of the extrinsic magnetic field (**B**) on the transmittance spectra of the 1D *GaAs/AlAs* ternary PC. The application of **B** (in Equation (5)) alters the dielectric response of the entire multilayer structure, thus changing the resonances of various modes. As **B** increases from 0 T to 0.75 T, the resonance frequency for the TE mode shifts from approximately 0.30 THz to 0.38 THz, while that of the TM mode shifts from about 0.29 THz to 0.35 THz. In addition to this frequency blue-shift, higher magnetic field strengths introduce additional mini-gaps and broaden the overall photonic band gap region. These findings highlight the extrinsic magnetic field as a powerful external control parameter for dynamically tuning both the position and number of band gaps against the propagation modes. Such magnetically tunable responses are particularly attractive for designing reconfigurable magneto-optical devices, including isolators, switches, and frequency-selective filters.

### 3.3. Effect of Electron Density ($n_{e}$)

**Figure 4:**
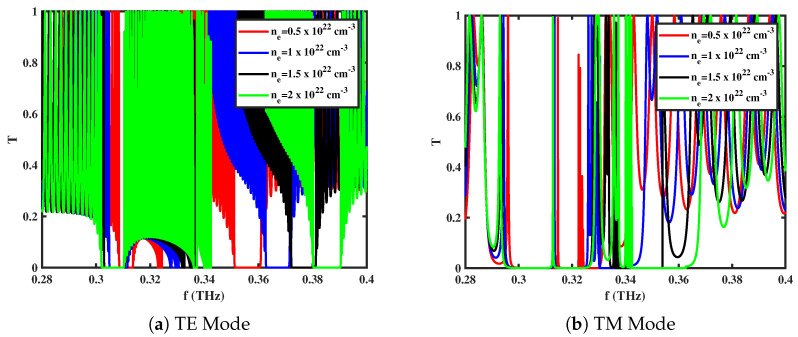
Transmittance spectra of *GaAs/AlAs*-based 1D ternary multilayered photonic crystal for different values of electron density ($n_{e}$) with **B** = 0.75 T and $\theta_{0}=0^{\circ }$ in different modes of propagation.

Figure 4a,b demonstrate the variation in the transmission spectrum of the 1D *GaAs/AlAs* ternary photonic structure with magnetized *GaAs* under different electron densities ($n_{e}$). As $n_{e}$ increases, the plasma frequency of the magnetized *GaAs* layers shifts, which in turn alters the location of the propagation modes around the structure of the photonic band. Specifically, an increase in the electron density causes the localized-mode resonances associated with both TE and TM polarizations to shift toward higher frequencies, accompanied by a slight broadening of the band gaps. This trend is attributed to the stronger interaction between the incident electromagnetic wave and the free carriers at a higher $n_{e}$. Consequently, the tunability of transmission characteristics through electron density adjustment provides a practical mechanism for controlling the spectral position and bandwidth of photonic band gaps, enabling the design of reconfigurable wide-band transmission filters.

### 3.4. Effect of Incident Angle ($\theta_{0}$)

**Figure 5:**
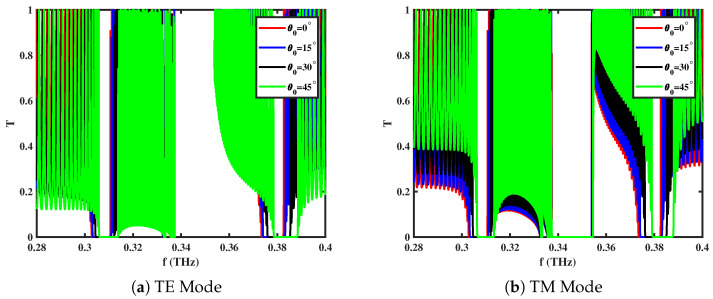
Transmittance spectra of *GaAs/AlAs*-based 1D ternary multilayered PC for different values of incident angle ($\theta_{0}$) with **B** = 0.75 T and $n_{e}=1.5\times 10^{22}$ cm^−3^ in different modes of propagation.

Figure 5a,b present the influence of the incident angle ($\theta_{0}$) on the transmittance spectra of the 1D *GaAs/AlAs* ternary PC under the action of an external magnetic field. As the incident angle increases from normal incidence ($0^{\circ }$) to $45^{\circ }$, the propagation-mode resonances shift noticeably. At normal incidence ($\theta_{0}=0^{\circ }$), the transmission spectra of the TE and TM waves overlap, showing no significant difference in their behavior. This is expected because, at normal incidence, both polarizations experience the same effective optical path, and the boundary conditions at each layer interface remain symmetric for electric and magnetic fields. However, as the incident angle increases, the effective refractive index experienced by the wave changes differently for the TE and TM polarizations due to the angular dependence of Snell’s law. This leads to a shift to the right (blue) in the PBG frequencies with increasing $\theta_{0}$. At the same time, the peak magnitudes remain unchanged, as the resonance condition still satisfies impedance matching at the externally magnetized layers. Such angle-dependent behavior is highly advantageous for designing polarization-sensitive and angularly selective photonic devices.

**Figure 6:**
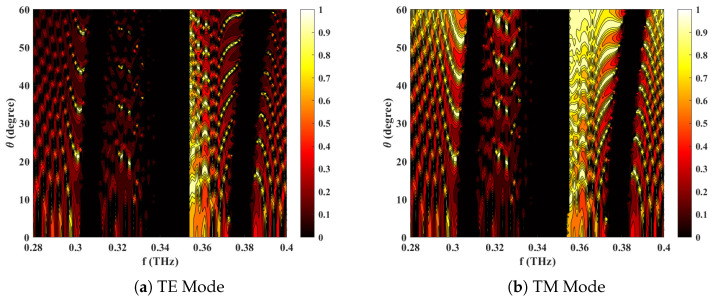
Numerically simulated transmittance for *GaAs/AlAs*-based 1D ternary multilayered PC as a function of frequency and incident angle (θ) in different modes of propagation with **B** = 0.75 T and $n_{e}=1.5\times 10^{22}$ cm^−3^.

The results presented in Figure 5a,b are further validated by the contour plot in Figure 6, which depicts the transmittance as a function of the incident angle and frequency. The transmittance bar clearly reveals multiple band gaps for symmetric 1D PCs while an external magnetic field is applied. Similar observations are also analyzed in Figure 5a,b, where the PBGs exhibit a systematic rightward shift (blue) with an increasing incident angle, a behavior attributed to the change in the effective propagation constant with the angle. Furthermore, the PBGs under TM polarization appear sharper and more distinct than those in the TE case. This arises from the stronger coupling between the parallel electric field component (under TM polarization) and the external magnetic layer resonance, which enhances mode separation and visibility across the photonic band structure.

## 4. Conclusions

This paper presents an investigation of the magneto-optical response of a one-dimensional *GaAs/AlAs* ternary photonic crystal including an extrinsically magnetized *GaAs* layer. The applied magnetic field gives rise to a pronounced tunability of the photonic band structure, as was analyzed from the numerical results. Correspondingly, with an increase in the external magnetic field up to 0.75 T, the central photonic band gap shifts from 0.30 to 0.38 THz, showing strong magnetic field-induced modulation. The calculated transmission spectra demonstrate a modulation depth of nearly 100% (complete PBG), proving the great sensitivity of the structure to the magneto-optical variation in *GaAs*. In addition, it follows that the frequency of the modes clearly shifted; it increased from 0.30 to 0.36 THz or 0.38 THz under an applied field, with participation of anisotropic permittivity in producing the splitting of a localized mode. These quantitative results confirm that the mechanism of extrinsic magnetization provides an effective method for dynamic control over band gaps and defect states without geometrical structure reconfiguration; therefore, the proposed ternary configuration could be promising for compact tunable photonic components.

In general, these results confirm that extrinsic parameters provide effective mechanisms to engineer the spectral response of 1D photonic crystals. The observed tunability and polarization sensitivity make such structures promising candidates for the development of reconfigurable, wide-band transmission filters and advanced magneto-optical devices.

## Figures and Tables

**Table 1:** Numerical values used for the simulation.

Parameters	Values
External magnetic field (**B**)	0.75 T
Electron concentration ($n_{e}$)	$1.5\times 10^{22}$ cm^−3^
Static permittivity ($\epsilon_{s}$)	12.9
Angle of incident ($\theta_{0}$)	$0^{\circ }$
Electron mass ($m_{e}$)	$9.1\times 10^{-31}$ kg
Effective mass of electron ($m^{*}$)	0.066 m
Vacuum permittivity ($\epsilon_{0}$)	$8.854\times 10^{-12}\;$C^2^N^−1^m^−2^
Spacial periodicity (*N*)	30
*GaAs* ($d_{G}$)	60 μm
*AlAs* ($d_{A}$)	100 μm
Magnetized *GaAs* ($d_{M}$ )	110 μm
Plasma frequency ($\omega_{p})$	0.707 THz

## Data Availability

The original contributions presented in this study are included in the article. Further inquiries can be directed to the corresponding authors.
